# Role of mTORC1 signaling in postnatal microglia activation preceding neurodegeneration in a mouse model for Niemann-Pick disease Type C

**DOI:** 10.1371/journal.pone.0330437

**Published:** 2025-09-05

**Authors:** Caroline E. Murray, David S. Betancourt-Trompa, Michael S. Martinez, Julia M. Carson, Lindsay J. Walsh, Will C. Remillard, Kayla B. Fordyce, Rohan Reddy, Ileana Soto

**Affiliations:** 1 Department of Biology, Providence College, Providence, Rhode Island, United States of America; 2 Department of Biology, Brown University, Providence, Rhode Island, United States of America; Sapienza University of Rome, ITALY

## Abstract

In *Npc1* deficient mice, postnatal developmental alterations in cerebellar microglia and Purkinje cells (PCs) are followed by early-onset neurodegeneration. Even in the absence of PC loss, microglia in *Npc1*^*nmf164*^ mice display hallmark features of activation during early postnatal development, including increased proliferation, enhanced phagocytic activity, and morphological changes indicative of an activated state. In this study, we investigated whether mammalian target of rapamycin complex 1 (mTORC1) drives postnatal activation of cerebellar microglia in *Npc1*^*nmf164*^ mice. We found that elevated CLEC7A (Dectin-1) expression and phosphorylation of S6 ribosomal protein (pS6R), a downstream target of mTORC1, co-occurred in microglial precursors within the developing white matter region (dWMR) of wild-type (WT) mice at postnatal day 7 (P7), as well as in neurodegeneration-associated microglia located in the molecular layer (ML) of *Npc1*^*nmf164*^ mice at P60. In contrast, microglia in the WMR of *Npc1*^*nmf164*^ mice at P60 did not show evidence of CLEC7A expression or increased mTORC1 activation. Interestingly, microglial precursors in the dWMR of *Npc1*^*nmf164*^ mice did not exhibit increased mTORC1 activation at P7 but instead showed delayed increased activation at P10. Inhibiting mTORC1 signaling with rapamycin from P10 to P21 reduced both microglial proliferation and soma size in *Npc1*^*nmf164*^ mice. Additionally, rapamycin treatment preserved VGLUT2⁺ presynaptic terminals/axons that innervate PC dendrites and decreased the total volume of CD68⁺ phagosomes per microglial cell, suggesting a reduction in phagocytic activity. However, the volume of VGLUT2⁺ synaptic material per phagosome remained unchanged between vehicle- and rapamycin-treated groups. While rapamycin enhanced myelination in *Npc1*^*nmf164*^ mice, it did not alter microglial phenotypes in the cerebellar WMR, suggesting that mTORC1 signaling does not mediate WMR microglial activation in this model. Together, our findings demonstrate that mTORC1 activation contributes to the aberrant activation of postnatal ML microglia and to early cerebellar pathology in *Npc1*^*nmf164*^ mice.

## Introduction

Recessive mutations in the *Npc1* or *Npc2* genes cause Niemann-Pick disease Type C (NPC), a lysosomal storage disease that lead to the disruption of lysosomal cholesterol transport and accumulation [1]. Although, the onset of NPC can occur at any age, most patients present with neurological symptoms between middle to late childhood, including early signs of cerebellar dysfunction such as clumsiness, vertical gaze palsy, gait disturbances, and eventually ataxia [1]. Intriguingly, many NPC patients are born apparently healthy and asymptomatic during infancy but progressively develop neurological symptoms during childhood. Neurologic symptoms include developmental regression, cognitive impairment, and dementia before an early death at childhood or juvenile stages [1]. In late-infantile and juvenile onset forms of NPC, important questions remain regarding which developmental processes in the brain are disrupted by NPC1 deficiency prior to the onset of early neurodegeneration and symptomatic stages in childhood.

Using the late-onset *Npc1*^*nmf164*^ mouse model, we previously found that loss of PCs is detected at postnatal day 60 (P60), but not at P30. At P60, strong activation of neurodegeneration-associated microglia in the ML along with the engulfment of dendrites from degenerating PCs is remarkable and distinctly from other regions of the cerebellum [2]. However, microglia proliferation and activation are already found prior to neurodegeneration at P30, raising the question if early changes in microglia are associated with developmental disturbances caused by NPC1 deficiency [2]. In fact, during early stages of cerebellar postnatal development, significant changes in the migration, proliferation, and differentiation of microglia precursors precede neurodegeneration of PCs in *Npc1*^*nmf164*^ mice [3–5]. Increased phagocytic activity and accumulation of phagosomes in differentiating microglia also occur in NPC1 deficient cerebella. The significant role of activated microglia in the progression of PCs degeneration in NPC has been well documented [2,6], with the increased life span of Npc1^-/-^ mice when microglia activation is genetically inhibited in these mice [6]. Furthermore, microglia deficient of NPC1 exhibit molecular signatures associated with the disease associated microglia (DAM) phenotype even in the absence of PC degeneration [4,6], suggesting that autonomous NPC1 deficiency impacts microglia identity and function. Nevertheless, the mechanisms by which lack of NPC1 shifts the identity of homeostatic microglia to a DAM phenotype, and how early this shift occurs, remain unknown.

Based on a specific molecular signature, a subpopulation of developmental microglia was previously identified as proliferative region associated microglia (PAM) in healthy mice [7]. These PAM, which are abundantly located in the developing WMR of the cortex and cerebellum of P7 mouse pups, share the gene signature of degenerative disease-associated microglia (DAM phenotype). The expression of genes like *Clec7a, Spp1, Apoe,* and *Gpnmb* is temporally and regionally limited to PAM in the developing WMR of the cerebellum, where these cells phagocytose myelin and newly formed oligodendrocytes at P7 [7]. It has been speculated that PAM population is not a universal intermediate step for microglia development, instead they are a subset of microglia transcriptionally pre-disposed to favor phagocytosis or a transient phenotypic behavior that is triggered by phagocytosis challenges (e.g., neurodegeneration) [7]. Interestingly, a marked increase of CLEC7A^+^ cells is observed in the cerebella of *Npc1*^*nmf164*^ mice at P10 [3], suggesting that during early postnatal development, NPC microglia may be further adopting or expanding a phagocytic identity, such as that associated with DAM. This highly phagocytic microglial cell in NPC could be more damaging for PCs as suggested by our and others previous work [2,4–6].

Because NPC1 deficiency in other type of cells affects metabolism through the hyperactivation of the mTORC1 pathway [8,9], and activation of this pathway is associated with activated microglia [10–12], we aimed to study the impact of this pathway in the postnatal development of NPC1 deficient microglia. We hypothesized that hyperactivation of mTORC1 could promote an immature state and contribute to the manifestation of the DAM phenotype in *Npc1*^*nmf164*^ microglia at postnatal stages before neurodegeneration occurs. To determine if the mTORC1 lysosomal-mediated metabolism is disrupted in developmental microglia and leads to changes in microglia identity and function in NPC, we used WT-*Tmem119*^*EGFP*^ and *Npc1*^nmf164^-*Tmem119*^*EGFP*^ mice where EGFP expression is driven by the *Tmem119* gene promoter. Expression of the *Tmem119* gene, a microglia-specific marker, is associated with the adult homeostatic state; therefore, we first validated EGFP fluorescence as a readout of mature homeostatic microglia in wild type mice as suggested previously by others [13,14]. Using these transgenic mice, we examined *Tmem119*^*EGFP*^ and CLEC7A expression, assessed proliferation, and cell morphology, and characterized the temporal and spatial changes in microglia at the early postnatal stage P7. Prior to assessing mTORC1 activation in postnatal microglia, we first confirmed the co-occurrence of mTORC1 pathway activation and CLEC7A re-expression in activated microglia during the neurodegenerative stage P60 in the cerebella of *Npc1*^nmf164^ mice. Similarly, we found that CLEC7A expression and mTORC1 pathway activation also co-occur in normal developmental microglia during early postnatal stages (P7). However, in *Npc1*^nmf164^ mice, the increased activation of mTORC1 was primarily detected at P10 instead that at P7, suggesting a delayed activation of this pathway in NPC1 deficient postnatal microglia. Using rapamycin to inhibit mTORC1 in *Npc1*^nmf164^ mice during postnatal development, we found the impact of this pathway in postnatal microglia and the developmental pathology associated with NPC1 deficiency in *Npc1*^nmf164^ mice. Our results suggest that the mTORC1 pathway contributes to the activated microglial phenotype observed at postnatal stages and prior to early neurodegeneration in *Npc1*^nmf164^ mice.

## Results

### Validating *Tmem119*^EGFP^ expression as a marker of maturing microglia in wild-type mice at postnatal day 7

**Fig 1:**
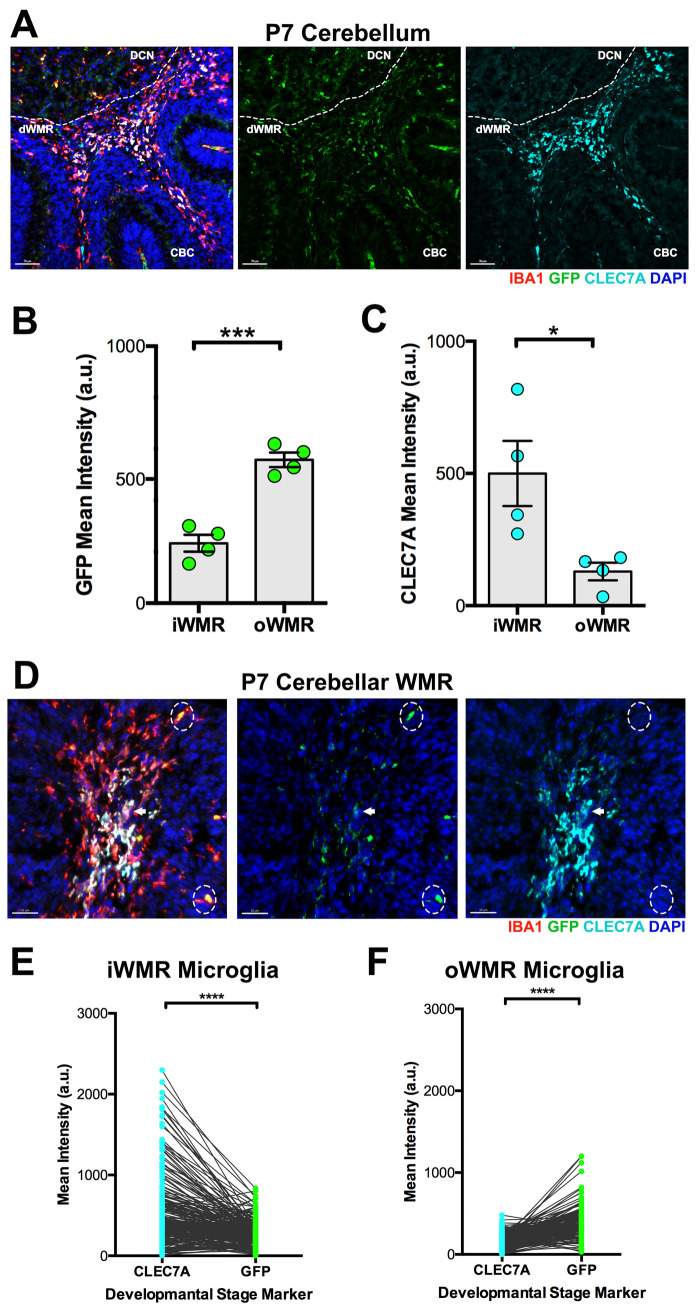
The expression of *Tmem119*^EGFP^ is lower in CLEC7A expressing microglia at P7. A. Cerebellum from a *Tmem119*^EGFP^ mouse immunolabeled with IBA1 and CLEC7A at P7. B. Quantitative analysis of the *Tmem119*^EGFP^ mean intensity inside (iWMR) and outside (oWMR) of the WMR. C. Quantitative analysis of the CLEC7A immunofluorescence mean intensity in iWMR and oWMR at P7. D. High-magnified image of IBA1 and CLEC7A Immunoreactivity in the cerebellar WMR of a *Tmem119*^EGFP^ mouse at P7. Most IBA1^+^ cells in the WMR show low expression of *Tmem119*^EGFP^ and high levels of CLEC7A (white arrow). IBA1^+^ cells outside the WMR (dashed line circles) show high levels of *Tmem119*^EGFP^ and low or no levels of CLEC7A. E. Quantitative paired analysis of the *Tmem119*^EGFP^ and CLEC7A mean fluorescence intensity in individual cells within the WMR. F. Quantitative paired analysis of the *Tmem119*^EGFP^ and CLEC7A mean fluorescence intensity in individual cells outside the WMR. Data are presented as mean ± SEM, (B and C) n = 4 mice, (E and F), n = 247 cells iWMR and176 cells oWMR from 4 mice/group. *P < 0.05, **P < 0.01, ***P < 0.001, ****P < 0.0001 by unpaired *t*-test (B-C) and paired *t*-*t*est (E-F). Scale bar: (A) 70 μm (D) 30 µm.

Before using the *Npc1*^nmf164^-*Tmem119*^*EGFP*^ mice to study the effects of NPC1 deficiency on postnatal microglial maturation and activation, we first examined the correlation between CLEC7A and *Tmem119*^EGFP^ expression in wild-type PAM precursors in the cerebellum at P7. Previous studies have shown transient upregulation of *Clec7a* expression in PAM precursors in the developing wild-type WMR at P7 [7]. To determine if *Tmem119*^EGFP^ is a reliable marker of microglia maturity and confirm *Clec7a* expression as a signature of immature microglia, immunofluorescence experiments with IBA1 (myeloid and microglial cells) and CLEC7A were performed in *Tmem119*^EGFP^ mice at P7. Our results show that the mean fluorescence intensity of *Tmem119*^*EGFP*^ in IBA1⁺ cells within the developing white matter region (iWMR) was significantly lower than in IBA1⁺ cells located outside this region (oWMR), which includes the deep cerebellar nuclei (DCN) and the developing cerebellar cortex (CBC) (Fig 1A–B). Consistent with previous reports [7], CLEC7A fluorescence intensity was significantly higher and predominantly localized to wild-type IBA1⁺ microglia within the WMR at this postnatal stage (Fig 1A–C). Notably, microglia with high CLEC7A expression exhibited low levels of *Tmem119*^*EGFP*^, and conversely, microglia with high *Tmem119*^*EGFP*^ expression showed low CLEC7A levels (Fig 1D–E). As a result, no CLEC7A immunoreactivity was detected in IBA1⁺ microglia of the DCN or CBC (oWMR), which predominantly express high levels of *Tmem119*^*EGFP*^. These findings confirm that CLEC7A is primarily expressed by immature PAM within the dWMR at P7 and *Tmem119*^*EGFP*^ is highly expressed by maturing microglia (Fig 1A–F).

### mTORC1 activation coincides with CLEC7A upregulation in developmental and neurodegeneration-associated microglia in *Npc1*^*nmf164*^ mice

**Fig 2:**
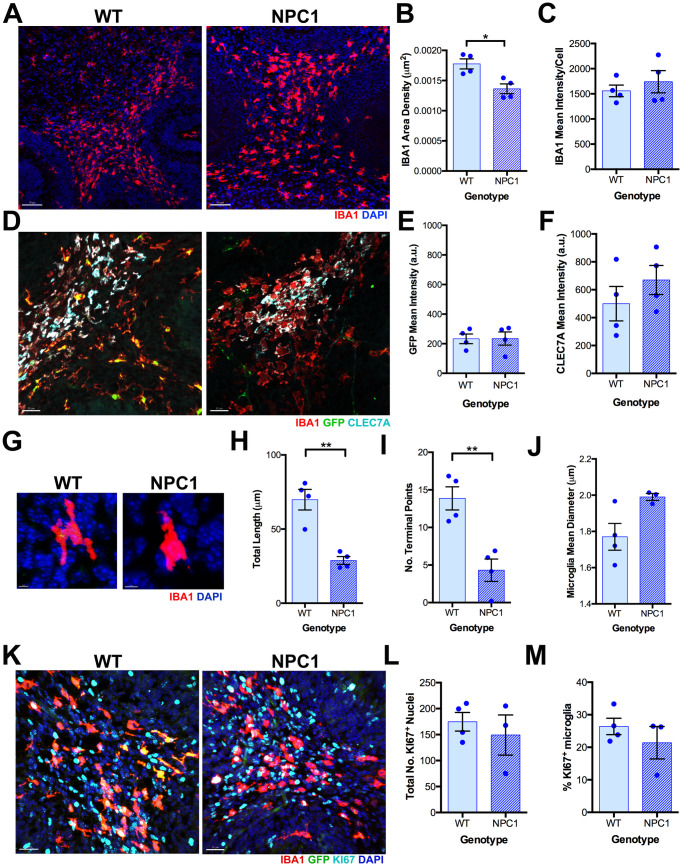
NPC1 deficiency in WMR microglia promotes a more ameboid shape phenotype at P7. A. IBA1 immunoreactivity in cerebella from WT and *Npc1*^*nmf164*^ mice at P7. B. Quantitative analysis of the density of IBA1^+^ cells in WT and *Npc1*^*nmf164*^ mice at P7. C. Quantitative analysis of the IBA1 immunoreactivity mean intensity per cell in WT and *Npc1*^*nmf164*^ mice at P7. D. IBA1 and CLEC7A immunoreactivity and *Tmem119*^EGFP^ expression in cerebella from *Tmem119*^EGFP^-WT and *Tmem119*^EGFP^*-Npc1*^*nmf164*^ mice at P7. E. Quantitative analysis of *Tmem119*^EGFP^ mean intensity per IBA1^+^ cell in WT and *Npc1*^*nmf164*^ mice at P7. F. Quantitative analysis of CLEC7A mean intensity per IBA1^+^ cell in WT and *Npc1*^*nmf164*^ mice at P7. G. Representation of the morphological structure of IBA1^+^ PAM cells in WT and *Npc1*^*nmf164*^ mice at P7. H. Quantitative analysis of dWMR IBA1^+^ cells total length in WT and *Npc1*^*nmf164*^ mice at P7. I. Quantitative analysis of dWMR IBA1^+^ microglia terminal points in WT and *Npc1*^*nmf164*^ mice at P7. J. Quantitative analysis of dWMR IBA1^+^ microglia processes mean diameter in WT and *Npc1*^*nmf164*^ mice at P7. K. Cerebella from *Tmem119*^EGFP^*-*WT and *Tmem119*^EGFP^*-Npc1*^*nmf164*^ mice at P7 immunolabeled with IBA1 and KI67. L. Quantitative analysis of the percentage of KI67^+^/IBA1^+^ microglia in the dWMR of *Tmem119*^EGFP^*-*WT and *Tmem119*^EGFP^*-Npc1*^*nmf164*^ cerebella at P7. M. Quantitative analysis of the total number of KI67^+^ nuclei within the dWMR of *Tmem119*^EGFP^*-*WT and *Tmem119*^EGFP^*- Npc1*^*nmf164*^ cerebella at P7. Data are presented as mean ± SEM, n = 3-4 mice. *P < 0.05 and **P < 0.01 by unpaired *t*-tes*t*. Scale bar: (A) 70 μm, (D and K) 30 μm, (G) 5 μm.

Given that lack of NPC1 can induce metabolic changes through the hyperactivation of the metabolic regulator mTORC1 [8], our next question was whether postnatal differentiation of PAM cells could be impaired in NPC1 deficient mice. First, we examined whether microglia precursors in *Npc1*^*nmf164*^ mice showed developmental changes at P7 when compared to wild-type cells. In *Npc1*^*nmf164*^ mice at P7, IBA1^+^ cells within the dWMR appeared more dispersed, leading to a significant reduction in their density compared to wild-type mice (Fig 2A–B). These NPC1 deficient cells had similar mean fluorescence intensity for IBA1 immunoreactivity, *Tmem119*^EGFP^ fluorescence, and CLEC7A immunoreactivity compared to wild-type cells at P7 (Fig 2C–F). However, at this postnatal stage, significant changes in microglia morphology were found in IBA1^+^ PAM within the dWMR of *Npc1*^*nmf164*^ mice compared to wild-type mice, including decreased total length and number of terminal points (branching) but not mean processes diameter (thickness) (Fig 2G–J), indicating a less ramified and more ameboid microglial phenotype. Additionally, no significant differences were observed in overall cell proliferation, measured by KI67 immunoreactivity, a widely used marker of active cell cycling, or in the percentage of IBA1⁺ microglia co-expressing KI67 between wild-type and *Npc1*^*nmf164*^ mice in the cerebellar dWMR at P7 (Fig 2K–M).These data suggest that NPC1 deficiency affects primarily the morphology and maturation of PAM cells at P7.

**Fig 3:**
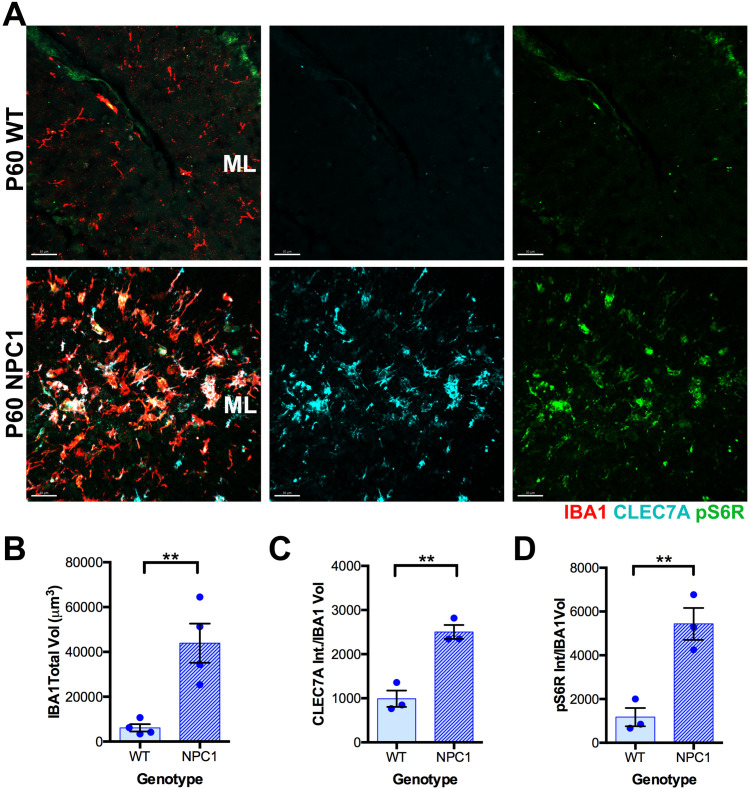
CLEC7A and pS6R immunoreactivity is increased in *Npc1*^*nmf164*^ microglia at P60. A. IBA1, CLEC7A, and pS6R immunoreactivity in the cerebellar ML of WT and *Npc1*^*nmf164*^ mice at P60. B. Quantitative analysis of the IBA1 immunoreactivity total volume in WT and *Npc1*^*nmf164*^ mice. C. Quantitative analysis of the CLEC7A immunoreactivity total intensity per IBA1^+^ cell volume in WT and *Npc1*^*nmf164*^ mice. D. Quantitative analysis of the pS6R immunoreactivity total intensity per IBA1^+^ cell volume in WT and *Npc1*^*nmf164*^ mice. Data are presented as mean ± SEM, n = 4 mice/group, *P < 0.05, **P < 0.01 by un-paired *t*-tes*t*. Scale bar: (A) 30 μm.

Previously, we showed that CLEC7A is increased in microglia at the ML during the degenerative stages of PCs in the *Npc1*^*nmf164*^ mouse model [3]. Given that CLEC7A expression is a shared feature of both developmental and DAM microglia in NPC1 deficient mice [3,6,7], and that mTORC1 activation is linked to activated microglial states [10–12], we sought to confirm whether mTORC1 is activated in *Npc1*^*nmf164*^ microglia at a neurodegenerative stage (P60). By examining the phosphorylation of the mTORC1 pathway substrate S6R in the cerebella of *Npc1*^*nmf164*^ mice at P60, we found that NPC1 deficient microglia in the ML significantly increased IBA1, CLEC7A, and pS6R immunoreactivity compared to wild-type mice (Fig 3A–D). However, although the total volume of IBA1^+^ microglia at the WMR was significantly increased in *Npc1*^*nmf164*^mice compared to wild-type mice at P60 (S1A and B Fig), no significant differences in pS6R fluorescence intensity was found in these cells between ^*164mfn*^*1Npc* and wild-type mice (S1A and C Fig). Additionally, at this neurodegenerative stage (P60), CLEC7A immunoreactivity was not detected in wild-type or NPC1 deficient cells within the WMR. These data suggest that during PC degeneration in *Npc1*^*nmf164*^ mice, activated microglia in the ML are characterized by mTORC1 pathway activation, and elevated CLEC7A expression.

### mTORC1 activation is delayed but still increased in postnatal *Npc1*^*nmf164*^ microglia

**Fig 4:**
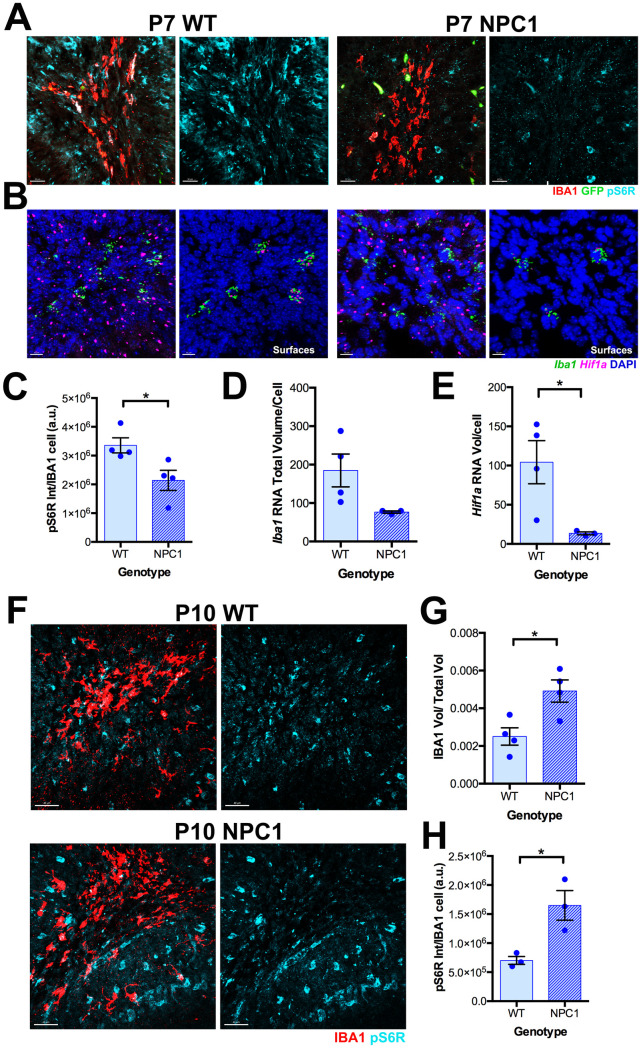
Changes in pS6R immunoreactivity and *Hif1a* expression in *Npc1*^*nmf164*^ mice. A. IBA1 and pS6R immunoreactivity in the dWMR from WT and *Npc1*^*nmf164*^ cerebella at P7. B. Expression of *Iba1* and *Hif1a* by RNAScope in the dWMR of WT and *Npc1*^*nmf164*^ cerebella at P7. C. Quantitative analysis of pS6R total fluorescence intensity per total IBA1^+^ cells volume in WT and *Npc1*^*nmf164*^ mice at P7. D. Quantitative analysis of *Iba1* mRNA total volume per cell in WT and *Npc1*^*nmf164*^ mice at P7. E. Quantitative analysis of *Hif1a* mRNA total volume per *Iba1* expressing cell in WT and **Npc*1*^*nmf164*^ mice at P7. F. IBA1 and pS6R immunoreactivity in cerebella from WT and *Npc1*^*nmf164*^ mice at P10. G. Quantitative analysis of IBA1 immunoreactivity total volume/image total volume in WT and *Npc1*^*nmf164*^ mice at P10. H. Quantitative analysis of the pS6R immunoreactivity total intensity per total IBA1 immunoreactivity volume in WT and *Npc1*^*nmf164*^ mice at P10. Data are presented as mean ± SEM, n = 3-4 mice/group, *P < 0.05, by unpaired *t*-*t*est. Scale bar: (A) 30 μm, (B) 10 μm, (F) 40 μm.

Given that pS6R immunoreactivity was increased along with CLEC7A immunoreactivity in neurodegeneration-associated microglia at the ML of *Npc1*^*nmf164*^ mice at P60, we interrogated if mTORC1 pathway was also activated in healthy PAM cells within the dWMR at P7, which highly express CLEC7A. Immunofluorescence experiments showed strong pS6R immunoreactivity in PAM cells and other non-microglial cells in the dWMR of wild-type mice at P7 (Fig 4A). Interestingly, at this postnatal stage (P7), the mean fluorescence intensity of pS6R was significantly reduced in IBA1^+^ PAM cells within the dWMR of *Npc1*^*nmf164*^ mice compared to wild-type controls (Fig 4A and C).

Activation of the mTORC1-S6K pathway in microglia can promote metabolic reprogramming through transcriptional, translational, and post-translational regulation of HIF1A signaling [15–17]. Using RNAscope, we assessed *Hif1a* mRNA expression in *Iba1*⁺ microglia. We found that the expression of *Iba1*, *Clec7a*, and *Hif1a* mRNA was simultaneously and significantly elevated in wild-type PAM at P7 and in neurodegeneration-associated microglia in the ML of *Npc1*^*nmf164*^ mice at P60, compared to age-matched wild-type ML microglia (S2A–D Fig). Analyzing *Npc1*^*nmf164*^ mice at P7, we found that although *Iba1* mRNA expression was slightly higher in wild-type PAM than in *Npc1*^*nmf164*^ PAM at P7, the difference did not reach statistical significance (Fig 4B and D). Like pS6R, *Hif1a* mRNA expression was also significantly decreased in *Iba1* expressing PAM within the dWMR in *Npc1*^*nmf164*^ mice compared to wild-type mice at P7 (Fig 4B and E), suggesting that lack of NPC1 decreases the activation of the mTORC1 pathway at this postnatal stage. However, analysis of P10 mice revealed that at this postnatal stage, both the total volume of IBA1^+^ cells and mean fluorescence intensity of pS6R were increased in *Npc1*^*nmf164*^ PAM compared to wild-type (Fig 4F–H). Given that levels of CLEC7A^+^ microglia are increased in the cerebellar WMR of *Npc1*^*nmf164*^ mice at P10 [3], our data suggest that increased mTORC1 activation occurs concurrently with the increased expression of CLEC7A.

### Rapamycin reduces proliferation and phagocytic activity in *Npc1*^*nmf164*^ microglia while enhancing synapses and myelination

**Fig 5:**
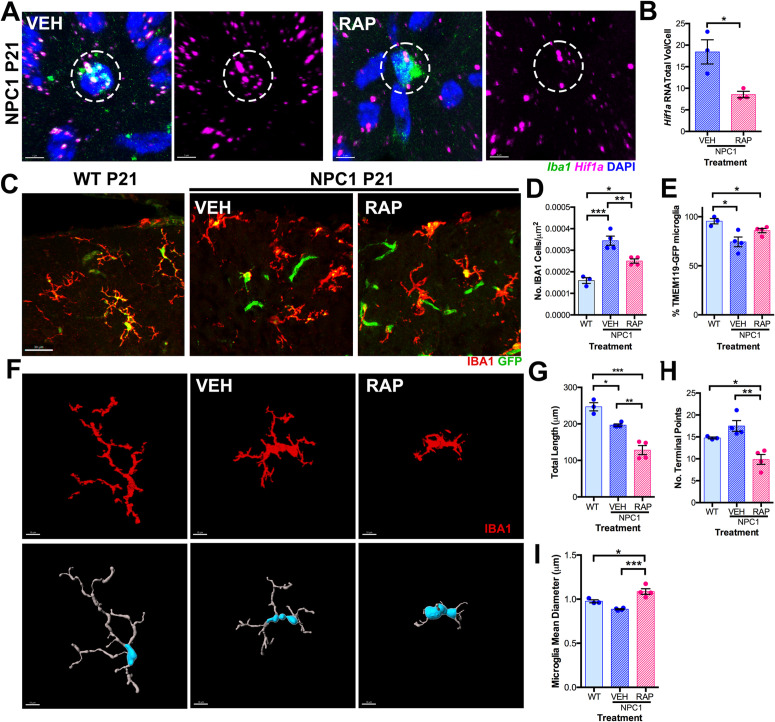
Effects of rapamycin treatment in *Npc1*^*nmf164*^ microglia at P21. A. Expression of *Iba1* and *Hif1a* mRNA by RNAScope in the cerebellar ML of VEH or RAP treated *Npc1*^*nmf164*^ mice at P21. B. Quantitative analysis of *Hif1a* mRNA total volume per *Iba1* mRNA expressing cell in VEH or RAP treated *Npc1*^*nmf164*^ cerebellar ML at P21. C. IBA1 immunoreactivity and *Tmem119*^EGFP^ expression in microglia at the ML of *Tmem119*^EGFP^-WT and VEH or RAP treated *Tmem119*^EGFP^*-Npc1*^*nmf164*^ mice at P21. D. Quantitative analysis of the number of IBA1^+^ cells in the cerebellar ML of WT and VEH and RAP treated *Npc1*^*nmf164*^ mice at P21. E. Quantitative analysis of the percentage of *Tmem119*^EGFP+^ microglia in the cerebellar ML of WT and VEH or RAP treated *Npc1*^*nmf164*^ mice at P21. F. Representation of the morphological structure of ML IBA1^+^ microglia in WT and VEH or RAP treated *Npc1*^*nmf164*^ mice at P21 (upper row-IBA1 immunoreactivity and lower row- filament tracing). G. Quantitative analysis of IBA1^+^ microglia total length in WT and VEH or RAP treated *Npc1*^*nmf164*^ mice at P21. H. Quantitative analysis of the number of IBA1^+^ microglia terminal points in WT and VEH or RAP treated *Npc1*^*nmf164*^ mice at P21. I. Quantitative analysis of the IBA1^+^ microglia mean diameter in WT and VEH or RAP treated *Npc1*^*nmf164*^ mice at P21. Data are presented as mean ± SEM, n = 3-4 mice/group, *P < 0.05, **P < 0.01, and ***P < 0.001 by unpaired *t*-test (B) or One-way ANOVA wi*t*h post-hoc Tukey. Scale bar: (A) 5 μm, (C) 30 μm, (F) 10 μm.

Increased proliferation of cerebellar CLEC7A^+^ microglial precursors in *Npc1*^*nmf164*^ mice at P10 along with increased phagocytic activity of maturing microglia at P14 suggest that lack of NPC1 significantly alters microglia development and phenotype [3]. Since we found increased activation of mTORC1 in *Npc1*^*nmf164*^ microglia at P10, we next tested if inhibition of the mTORC1 pathway by rapamycin treatment could prevent or modify the microglial developmental alterations caused by NPC1 deficiency. Lactating females were provided drinking water with 40ug/ml of rapamycin (RAP) or 0.5% ethanol as a vehicle (VEH) to treat *Npc1*^*nmf164*^ mouse pups through maternal lactation from P10 to P21. By postnatal day 21 (P21), microglial precursors are no longer present in the white matter region (WMR); many have migrated into the various layers of the cerebellar cortex, including the molecular layer (ML), where they have differentiated into more mature, homeostatic microglia [3,18,19]. As expected, postnatal rapamycin treatment of *Npc1*^*nmf164*^ mice significantly reduced *Hif1a* expression in *Iba1* expressing microglia at the ML compared to *Iba1* expressing microglia in vehicle treated *Npc1*^*nmf164*^ mice (Fig 5A–B). No differences in *Iba1* mRNA expression were found between vehicle- and rapamycin-treated *Npc1*^*nmf164*^ mice (VEH 44.55 ± 10.63 vs RAP 55.71 ± 8.35, n = 3, *P* value = 0.455). These results corroborated that rapamycin treatment inhibited the mTORC1-HIF1a pathway in these cells. Additionally, rapamycin treatment prevented the increase in the number of IBA1^+^ microglia in the cerebellar ML of *Npc1*^*nmf164*^ mice compared to vehicle treated *Npc1*^*nmf164*^ mice, although not down to the levels of wild-type controls (Fig 5C–D). However, the significant decrease in the percentage of IBA1^+^ microglia with high expression of *Tmem119*^EGFP^ in vehicle treated *Npc1*^*nmf164*^ mice compared to WT- *Tmem119*^EGFP^ mice was not prevented by rapamycin (Fig 5C and E). Microglia at the ML in vehicle-treated *Npc1*^*nmf164*^ mice showed a significant decrease in total length compared to wild-type mice (Fig 5F–G). However, a further significant reduction in total length and number of terminal points along with an increased mean processes diameter was found in rapamycin-treated *Npc1*^*nmf164*^ microglia when compared to wild-type and vehicle-treated *Npc1*^*nmf164*^ microglia (Fig 5F,H–I). These results suggest that inhibition of mTORC1 by rapamycin decreases proliferation and microglia cell growth in *Npc1*^*nmf164*^ mice.

**Fig 6:**
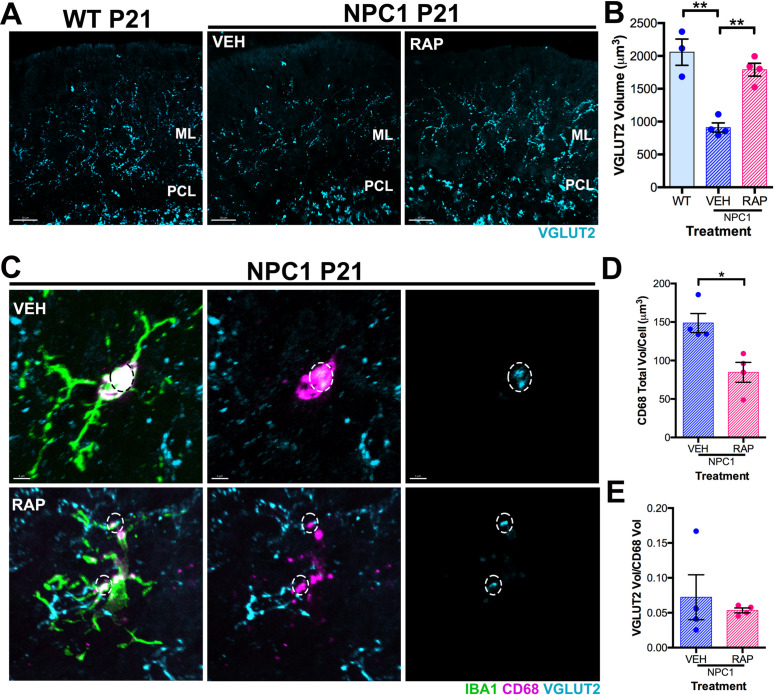
Rapamycin treatment prevents decrease of VGLUT2 presynaptic terminals/axons in *Npc1*^*nmf164*^ cerebellar ML. A. VGLUT2 immunoreactivity in WT and VEH or RAP *Npc1*^*nmf164*^ cerebellar ML at P21. B. Quantitative analysis of VGLUT2 immunoreactivity in cerebellar ML from WT and VEH or RAP treated *Npc1*^*nmf164*^ mice at P21. C. IBA1, CD68, and VGLUT2 immunoreactivity in representative ML microglia from WT and VEH or RAP *Npc1*^*nmf164*^ mice at P21. D. Quantitative analysis of the CD68 total volume per IBA1^+^ cell in WT and VEH or RAP treated *Npc1*^*nmf164*^ mice at P21. E. Quantitative analysis of the VGLUT2 volume per CD68 volume in WT and VEH or RAP treated *Npc1*^*nmf164*^ mice at P21. Data are presented as mean ± SEM, n = 3-4 mice, *P < 0.05 by unpaired *t*-*t*est. Scale bar: (A) 30 μm, (C) 5 μm.

VGLUT2^+^ presynaptic terminals/axons from climbing fibers, which innervate PC proximal dendrites, are significantly decreased in *Npc1*^*nmf164*^ mice at P14 [3]. Along with these findings, we also previously found an increased volume of these terminals engulfed by microglia in *Npc1*^*nmf164*^ mice compared to wild-type mice at P14. Here, we examined if rapamycin treatment alters the effects of NPC1 deficiency in VGLUT2^+^ presynaptic terminals/axons. We found that the significant decrease of VGLUT2^+^ in presynaptic terminals/axons observed in vehicle-treated *Npc1*^*nmf164*^ mice at P21 was prevented by rapamycin treatment when compared to wild-type mice (Fig 6A–B). We also found that the total volume of CD68^+^ phagosomes per microglia in the ML of *Npc1*^*nmf164*^ mice was significantly decreased by rapamycin compared to vehicle-treated *Npc1*^*nmf164*^ microglia (Fig 6C–D). However, no differences were found in the volume of engulfed VGLUT2^+^ presynaptic terminals/axons by microglial CD68^+^ phagosomes between vehicle- and rapamycin-treated *Npc1*^*nmf164*^ mice at this stage (Fig 6C and E). These data suggest that rapamycin treatment prevents the decrease of VGLUT2^+^ climbing fibers presynaptic terminals/axons and reduces the volume of microglial phagosomes in *Npc1*^*nmf164*^ mice during postnatal development potentially by decreasing phagocytic activity.

**Fig 7:**
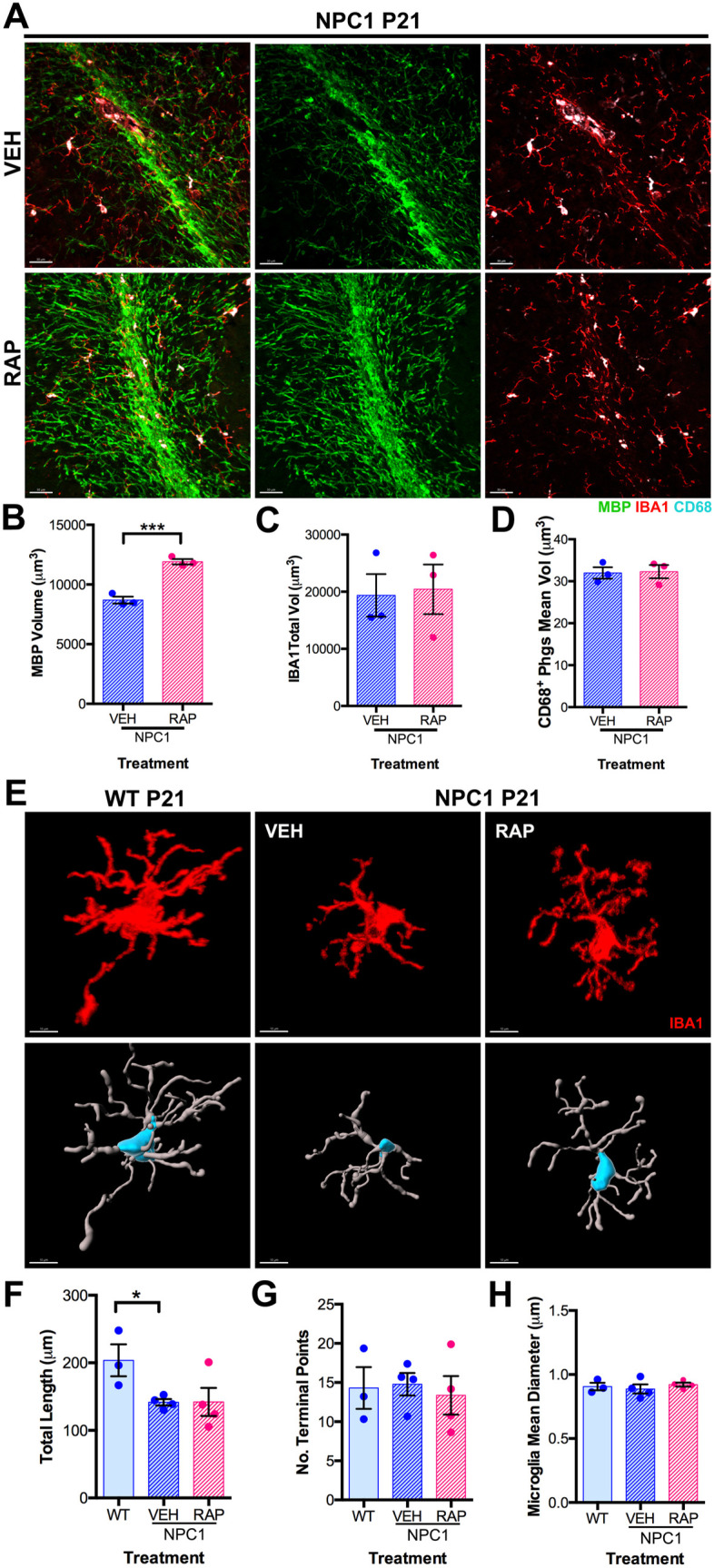
Rapamycin treatment enhances myelination in *Npc1*^*nmf164*^ cerebellum. A. MBP, IBA1, and CD68 immunoreactivity in the cerebellar WMR of VEH or RAP treated *Npc1*^*nmf164*^ mice at P21. B. Quantitative analysis of MBP total volume per image in the cerebellar WMR of VEH or RAP treated *Npc1*^*nmf164*^ mice at P21. C. Quantitative analysis of the IBA1 total volume per image in the WMR of WT and VEH or RAP treated *Npc1*^*nmf164*^ mice at P21. D. Quantitative analysis of the mean volume of CD68^+^ phagosomes in WT and VEH or RAP treated *Npc1*^*nmf164*^ mice at P21. F. Representation of the structure of IBA1^+^ microglia in the WMR of WT and VEH or RAP treated *Npc1*^*nmf164*^ mice at P21 (upper row-IBA1 immunoreactivity and lower row- filament). G. Quantitative analysis of IBA1^+^ microglia total length in the WMR of WT and VEH or RAP treated *Npc1*^*nmf164*^ mice at P21. H. Quantitative analysis of the number of IBA1^+^ microglia terminal points in the WMR of WT and VEH or RAP treated *Npc1*^*nmf164*^ mice at P21. I. Quantitative analysis of IBA1^+^ microglia mean processes diameter in the WMR of WT and VEH or RAP treated *Npc1*^*nmf164*^ mice at P21. Data are presented as mean ± SEM, n = 3-4 mice/group, *P < 0.05 and ***P < 0.001 by unpaired *t*-tes*t* (B-D) or One-way ANOVA with post-hoc Tukey (F-H). Scale bar: (A) 30 μm, (E) 10 μm.

It is known that myelination of PC axons is impaired in NPC1 deficient mice during postnatal development [20]. Additionally, it has been shown that microglia play an important role in developmental myelination [21]. Therefore, we examined the effect of rapamycin treatment in WMR microglia and the myelination of PC axons in *Npc1*^*nmf164*^ mice at P21. Using the myelin basic protein (MBP) antibody we found that rapamycin treatment significantly increased MBP immunolabeling in the cerebellar WMR of *Npc1*^*nmf164*^ mice compared to vehicle-treated *Npc1*^*nmf164*^ mice at P21 (Fig 7A–B). Yet, no differences in the total volume of IBA1^+^ microglia and the mean volume of CD68^+^ phagosomes were found between vehicle- and rapamycin-treated *Npc1*^*nmf164*^ mice (Fig 7A,C–D). Analysis of the morphological structure of WMR microglia showed that only total length, not the number of terminal points or mean processes diameter, was slightly but significantly decreased in vehicle-treated *Npc1*^*nmf164*^ cerebella compared to wild-type controls (Fig 7E–H). These data suggest that minimal changes in morphology occur in WMR microglia deficient of NPC1 when compared to wild-type microglia. Since no changes in pS6 immunoreactivity were observed in *Npc1*^*nmf164*^ microglia within the WMR, not even at the neurodegenerative stage P60 (S1 Fig), it is consistent that rapamycin treatment had no additional effects on these cells at P21.

## Discussion

During the normal postnatal development of the cerebellum, significant temporal and spatial cellular changes in microglial precursors including location, migration, proliferation, differentiation, and phagocytic activity can be identified between P0 and P21 [3,5,19,22,23]. At the postnatal stage when CLEC7A^+^ PAM are more abundant in the cerebellum (P7), microglial precursors are still migrating radially and differentiating [3,18,19], suggesting that migratory and differentiated PAM may contribute to the populations of mature microglia in other regions of the cerebellum (e.g., ML). Given that NPC1 deficiency leads to increased CLEC7A expression in dWMR microglia after postnatal day 7 and is associated with an activated postnatal microglial phenotype that precedes early PC degeneration in *Npc1*^*nmf164*^ mice [3], we sought to investigate the underlying mechanisms driving these pathological changes. There were two main questions in this study; first, if lack of NPC1 was impairing microglia maturation and expanding the PAM immature phenotype, which shares molecular and cellular features with the DAM phenotype. Secondly, we interrogated if NPC1 deficiency disrupts microglia cerebellar postnatal development through the dysregulation of the mTORC1 pathway. Although mixed-sex groups were used, due to limited sample sizes, all findings in this study were interpreted based on age and/or genotype differences alone.

To answer these questions, we used the *Tmem119*^EGFP^ mouse as a tool to identify maturing microglia. The expression of EGFP in *Tmem119*^EGFP^ mice correlates with the onset of microglial maturation and is significantly reduced in disease-associated or activated microglial states, such as those observed in neurodegeneration, where *Tmem119* is downregulated [13,14]. First, we corroborated that *Tmem119*^EGFP^ expression was associated with microglial maturation, as evidenced by its reduced fluorescence in CLEC7A^+^ PAM compared to CLEC7A^-^ microglia located in other cerebellar regions outside the WMR (oWMR) at P7. Strong expression of *Tmem119*^EGFP^ was also observed in mature microglia at juvenile stages (P21). Analyzing PAM cells in *Npc1*^*nmf164*^ mice at P7, we found that although these cells were more dispersed in the dWMR, no differences in IBA1, *Tmem119*^EGFP^, and CLEC7A expression and proliferation were found compared to wild-type mice. However, the morphology of *Npc1*^*nmf164*^ PAM was less ramified and rounded than wild-type PAM at P7.

Because lack of NPC1 induces the hyperactivation of the metabolic regulator mTORC1 [8], and hyperactivation of mTORC1 in mouse microglia *in vivo* leads to an ameboid shape morphology, increased proliferation, and phagocytic activity [12], we sought to determine the involvement of mTORC1 in NPC microglia pathology. Previous studies in *Npc1*^-/-^ mice [6] showed the significant increase of *Hif1a* mRNA expression in microglia, suggesting mTORC1 activation. To confirm the activation of mTORC1 in activated microglia during neurodegenerative stages, we examined pS6R immunoreactivity in P60 *Npc1*^*nmf164*^ cerebella. pS6 and CLEC7A immunoreactivity were absent in the ML of P60 wild-type mice but strongly present in IBA1 labeled activated microglia in the ML of *Npc1*^*nmf164*^ mice. The simultaneous surge of these markers in activated microglia suggests a correlation between mTORC1 activation and CLEC7A expression. Therefore, we examined pS6 immunoreactivity in dWMR-PAM cells in wild-type mice at P7 observing a remarkable pS6 immunolabeling in these cells. Furthermore, *Hif1a* mRNA expression was also high in wild-type PAM (P7) and *Npc1*^*nmf164*^ neurodegeneration-associated microglia (P60) co-expressing *Iba1* and *Clec7a.* mTORC1 can promote metabolic reprogramming through transcriptional, translational, and post-translational regulation of HIF1A signaling [15–17]. Increased expression of *Hif1a* is found in the DAM gene signature, and activation of microglia in Alzheimer’s disease mouse models is dependent on the mTORC1/HIF1A pathway, which leads to the glycolytic state [10]. Interestingly, CLEC7A is associated with the conversion of immunosuppressive macrophages (M2) to inflammatory macrophages (M1) by inducing the M1 metabotype [24], which is characterized by enhanced glycolytic metabolism and reduced mitochondrial activity [25]. Furthermore, activation of the mTORC1-HIF1a pathway is also associated with the induction of macrophages M1 [25]. Overall, these and our findings support a potential connection between CLEC7A expression and the activation of the mTORC1-HIF1a pathway which induces metabolic reprogramming in activated microglia.

In *Npc1*^*nmf164*^ mice, PAM cells exhibited increased pS6 immunoreactivity at postnatal day 10 (P10). This delayed upregulation paralleled the previously reported increase in CLEC7A expression in PAM at P10 in *Npc1*^*nmf164*^ mice [3], suggesting a temporally shifted or delayed microglial response to developmental cues. In fact, the natural appearance of PAM is concurrent with the start of myelination in the healthy cerebellum at P7 [7], therefore, it is possible that this delayed mTORC1 activation is due to the known disruption of myelination by NPC1 deficiency in mutant mice [26–28].

It is plausible that the normal metabolic transition from PAM to mature homeostatic microglia during postnatal microglial differentiation is disrupted in NPC. In support of this hypothesis, previous studies have shown that genetic hyperactivation of mTORC1 in mouse microglia *in vivo* leads to an ameboid shape morphology, increased proliferation, and phagocytic activity [12], features found in *Npc1*^*nmf164*^ cerebellar microglia during postnatal stages and prior neurodegeneration [2–5]. The delayed increase in mTORC1 activation in *Npc1*^*nmf164*^ PAM could lead to a temporal extension and/or spatial expansion of a more glycolytic/phagocytic microglia in subsequent postnatal stages. In fact, increased TREM2 expression, a protein involved in phagocytic function, is linked to increased mTORC1 activation in cortical microglia from *Npc1*^-/-^ mice at P21 [29]. In *Npc1*^*-/-*^ mice, *Hif1a* overexpression at the neurodegenerative stage ~P50 has been linked to an activated, glycolytic microglial phenotype [6]. To explore whether early mTORC1 activation contributes to altered microglial maturation, we inhibited the mTORC1 pathway from P10 to P21 in *Npc1*^*nmf164*^ mice using rapamycin. This treatment reduced microglial proliferation and *Hif1a* expression, indicating a role for mTORC1 in microglial activation. However, rapamycin also impaired microglial processes development, underscoring mTORC1’s importance in postnatal microglial growth and differentiation. The reduced percentage of ML microglia with high *Tmem119*^*EGFP*^ expression in *Npc1*^*nmf164*^ mice at P21 suggests persistent immaturity. However, rapamycin treatment failed to rescue this phenotype, indicating that mTORC1 signaling does not directly regulate *Tmem119* expression in this context.

Previous studies have shown that rapamycin treatment during neurodegenerative states, reduces microglia activation, ramification, and proliferation while decreasing the proinflammatory response [11,30], supporting the role of mTORC1 activation in the shift of microglia to a glycolytic activated state. Furthermore, mTORC1 activation in activated microglia has a significant role in phagocytosis, since rapamycin treatment decreases TREM2 expression in microglia and the clearance of β-Amyloid plaques in an Alzheimer’s disease mouse model [31]. At P14, *Npc1*^*nmf164*^ mice exhibit an increased number of microglial phagocytic cups in the ML, accompanied by a reduction in VGLUT2^+^ climbing fibers presynaptic terminals/axons, as well as enhanced microglial engulfment of these structures [3]. Here, rapamycin treatment significantly prevented the loss of VGLUT2^+^ presynaptic terminals/axons in *Npc1*^*nmf164*^ mice at P21. While the volume of VGLUT2 within CD68⁺ phagosomes was not different between *Npc1*^*nmf164*^ and wild-type mice, rapamycin markedly reduced the total volume of CD68⁺ phagosomes per microglial cell. These results may imply that mTORC1 inhibition decreased microglia phagocytic activity in *Npc1*^*nmf164*^ mice. A recent study also showed that rapamycin treatment or deletion of TREM2 in NPC1 deficient microglial cells *in vitro* reduces mTORC1 activation and phagocytic activity in microglia that included the reduction of CD68 protein levels [29]. The early mTORC1 overactivation in NPC1 deficient microglia could presumably increase the susceptibility of PCs to dysfunction and degeneration in NPC. However, it is also possible that rapamycin treatment exerts direct effects on neurons of the inferior olivary nucleus, which give rise to climbing fibers, thereby contributing to the preservation of VGLUT2 terminals/axons in *Npc1*^*nmf164*^ mice.

Finally, we found that rapamycin treatment increased myelination in PC axons but barely affected WMR microglia phenotype. It is known that NPC1 deficiency causes decreased myelination in PC axons, therefore, it is possible that rapamycin treatment had a direct effect on myelinating oligodendrocytes and precursors which are also affected by NPC1 deficiency [26–28]. Interestingly, despite reduced microglial processes length in P21 *Npc1*^*nmf164*^ mice, rapamycin did not alter WMR microglial features. Moreover, at the neurodegenerative stage (P60), WMR microglia lacked CLEC7A expression and showed no changes in pS6R immunoreactivity, suggesting they do not adopt a DAM phenotype. This is consistent with single-cell transcriptomic data showing that disease-associated, developmental, and injury-responsive microglia in demyelinating conditions share limited core genes but maintain distinct molecular signatures [32]. It is also possible that due to impaired myelin production in *Npc1*^*nmf164*^ mice, the microglial response to axonal degeneration in the WMR is weaker than in the ML.

## Materials and methods

### Animals

Experiments described here involving mice were conducted in accordance with policies and procedures described in the Guide for the Care and Use of Laboratory Animals of the National Institutes of Health and were approved by the Animal Care and Use Committees at Providence College. The results and experiments of this study that involves animals are also reported in accordance with ARRIVE guidelines. The C57BL/6J-*Npc1*^*nmf164*^/J mouse strain (Jax stock number 004817) was obtained from The Jackson Laboratory. *Npc1*^*nmf164*^ heterozygous mice were bred and housed in a 12/12-hour light/dark cycle to generate both wild-type and *Npc1*^*nmf164*^ homozygous mutant mice. We were also able to breed some *Npc1*^*nmf164*^ homozygous males with *Npc1*^*nmf164*^ heterozygous females to produce more *Npc1*^*nmf164*^ homozygous mice. To produce NPC1-deficient mice with microglia expressing GFP (*Npc1*^*nmf164*^*- Tmem119*^EGFP^), *Npc1*^*nmf164*^ heterozygous mice were intercrossed with the C57BL/6-*Tmem119*^*em2Gfng*^/J (Jax stock number 031823). The C57BL/6-*Tmem119*^*em2Gfng*^/J mouse uses the endogenous *Tmem119* promoter/enhancer sequences to direct the expression of enhanced green fluorescent protein specifically in microglia, while retaining the endogenous *Tmem119* expression [14]. Both males and females were used in this study, when using 4 mice, 2 females and 2 males were included per group.

### Rapamycin treatment

Rapamycin treatment was performed as previously described with some modifications [33]. Rapamycin (ThermoScientific Catalog number: J62473MF) was dissolved in 100% ethanol for a stock of 8 mg/ml; then further diluted to 40ug/ml in drinking water. Pregnant females heterozygous for *Npc1*^*nmf164*^ but homozygous for *Tmem119*^EGFP^, were assigned for rapamycin (RAP) or vehicle (VEH) group. At day 10 after the birth of the pups, the lactating females were provided with drinking water containing 40ug/ml of rapamycin, which was replaced weekly, for a final dose of 4 mg/kg/day. VEH group was provided with 1 mL of 100% ethanol in 200 mL of water, which was the solvent for the rapamycin stock. The lactating females with their litters were treated with rapamycin or vehicle up to 21 days after the birth of the pups (11 days). At P21, the pups were euthanized for tissue harvest as described below.

### Mouse perfusion and tissue preparation

For transcardiac perfusion, mice were euthanized with CO_2_, then perfused with 1X PBS followed by 4% paraformaldehyde. After dissection, brains were fixed by immersion in 4% paraformaldehyde overnight. Then brains were rinsed in 1X PBS, immersed in 30% sucrose/PBS solution overnight at 4°C, frozen in OCT, and cryosectioned as 40μm and 50μm floating sections.

### Immunohistochemistry

After collecting 40–50μm floating cryostat cerebellar sections, immunofluorescence experiments were performed as previously described [9]. Briefly, sections were collected in 1X PBS, rinsed once in 1X PBT (PBS + 1% Triton 100X), and incubated overnight at 4°C in a cocktail of primary antibodies that were diluted in 1X PBT + 20% normal donkey serum. After rinsing the tissue three times with 1X PBT, incubation with corresponding secondary donkey antibodies (1:500, Jackson-ImmunoResearch or Invitrogen) was followed. After 1.5 hours of secondary antibodies incubation, the cerebellar sections were washed, incubated with DAPI, and mounted in Poly-aquamount (Polysciences). The following primary antibodies were used: rabbit anti-IBA1 (1:200, Wako, # 019-19741), goat anti-IBA1 (1:200, GeneTex, #GTX89792), rat anti-CD68 (1:200, Bio-Rad, #MCA1957), guinea-pig anti-VGLUT2 (1:800, Synaptic Systems, # 135404), rabbit anti-phosphorylated S6R (1:200, Cell Signaling, #2211), rat anti-Dectin1 (CLEC7A) (1:100, 2A11, Bio-Rad, MCA2289).

### RNAScope

The RNAscope in situ hybridization assay was performed according to the manufacturer’s protocol provided by ACD Bio. Here, we briefly describe the key steps involved in the procedure. Adult mice were transcardiac perfused with buffered 4% PFA, brains were dissected and immersed in the same fixative solution overnight. Brains from P7 pups were fixed only by immersion using buffered 4% PFA. Cerebellar sections (16um) were fixed using 4% PFA in phosphate-buffered saline (PBS) for 1 hour at 4°C. After fixation, the sections were washed with PBS and were then dehydrated in a series of ethanol steps. Hydrogen peroxide was then applied for 10 minutes at room temperature to block endogenous peroxidases. Then the slides were washed in distilled water and treated with protease for 30 minutes at room temperature to permeabilize the tissues. Target probes obtained from ACD Bio and specific to the genes of interest (*Iba1 (catalog number: 319141)*, *Clec7a (catalog number: 532061-C3) and Hif1a (536761-C2*) were hybridized to the sections in a humidified chamber at 40°C for 2 hours using the HybEZ™ II Hybridization System (ACD Bio Catalog number: 323100). Following hybridization, signal amplification was carried out using the provided pre-amplifier and amplifier conjugated to alkaline phosphatase. For detection, the Tyramide Signal Amplification (TSA) system was employed to enable fluorescence detection of multiple probes simultaneously. Sections were incubated with fluorophore-conjugated tyramide reagents, which bind to the site of probe binding, amplifying the fluorescent signal. The cerebellar sections were washed, incubated with DAPI, and mounted in Poly-aquamount (Polysciences).

### Microscopy image analysis

All images were acquired using a Zeiss LSM 700 confocal microscope and analyzed with ImageJ or the Bitplane Imaris™ software. To keep consistency between samples, all imaging and quantitative analyses described here were performed in the first four anterior cerebellar lobules (I-IV). For quantification of IBA1+ and IBA1+/KI67+ cells in the developing cerebellum, one to two 40X images per cerebellar section and two cerebellar cryosections per mouse were used. The number of IBA1⁺ cells within equally sized regions of interest was manually quantified using ImageJ’s ‘Cell Counter’ tool. The total number of Ki67^+^ nuclei and Ki67^+^/IBA1^+^ cells in the WMR were calculated using Imaris software and manually counting the cells per image. For all the image analyses described below, investigators were blind to the genotype of the mice. To calculate the total volume of IBA1^+^ cells in the ML and WMR of P60 cerebellar sections, a Surface rendering of the IBA1^+^ cells was made for each 40X 3D image and the volume of the surfaces was calculated and provided by the Imaris software. The software also provided measurements of the mean or total fluorescence intensity of CLEC7A and pS6R per IBA1-defined surface (cell). For P7 samples, data were reported as total intensity per IBA1⁺ cell. However, due to the high density and clustering of microglia in the WMR at P10, fluorescence intensity for these markers was normalized to the total volume of IBA1⁺ cells per image rather than per individual cell.

For 3D image reconstructions and analyses the Bitplane Imaris™ software was used. RNA expression of *Iba1*, *Clec7a*, and *Hif1a* per cell in cerebellar sections was quantified using the *Cells* tool from Imaris, where the total volume of the RNAScope fluorescent grains surrounding the nucleus per cell was calculated for each gene probe. The total length, terminal points, and mean diameter of cerebellar postnatal microglia was quantified using the Filament Tracer plugin from the Imaris™ software. Two to three images per mouse (n = 3–4 mice) were used for the quantifications. Quantitative analysis of 3D images to determine the GFP and CLEC7A fluorescence mean intensity inside microglia was performed using the Imaris™ Surface rendering tool. First, IBA1^+^ microglia were segregated using the Surface rendering tool and the values for the mean intensity of each IBA1^+^ microglia for GFP and CLEC7A mean intensity were obtained from the software. The number of microglia positive for Tmem119^EGFP^ in the ML was quantified manually using Imaris.

For VGLUT2 quantification per cerebellar section, a region of interest of 500 μm X 250 μm Z = 30 was selected in the ML and a surface rendering for the VGLUT2 fluorescence was performed. The total volume of the VGLUT2 surfaces per cerebellar section was calculated and provided by the Imaris software. To quantify VGLUT2 synaptic terminals/axons engulfed by CD68^+^ phagosomes in microglia, a Surface rendering was made for CD68^+^ phagosomes, followed by the use of the “Mask all” tool to create a new channel for VGLUT2^+^ terminals/axons inside of the created surface (in this case CD68 surface) by clearing all the fluorescence that was not found overlapping with the CD68 rendering surface. A new Surface rendering was created for the masked VGLUT2^+^ terminals/axons inside the phagosomes and the volume of these surfaces were calculated by the software. Then the total volume of VGLUT2 per the volume of the CD68^+^ phagosome was calculated. Also, the volume of the surface rendering for IBA1^+^ microglial cells was also calculated in these images to calculate the CD68 total volume per IBA1^+^ cells. For the quantification of MBP immunofluorescence in 40X 3D images with Z = 13 from the WMR of the same lobe, a Surface rendering of MBP was made using Imaris and the value for the total volume of MBP per image was used.

### Statistical analysis

Data were analyzed using GraphPad Prism software. Significance was calculated using unpaired t-tests for comparisons between two groups or One-way ANOVA with post-hoc Tukey for comparisons of three groups. p-values are provided as stated by GraphPad Prism software and significance was determined with p-values less than 0.05.

## Supporting information

S1 Fig The intensity of pS6R immunofluorescence per IBA1 cell volume is decreased in WMR microglia from NPC1 deficient mice at neurodegenerative stage (P60). A. IBA1 and pS6R immunoreactivity in WMR microglia from WT and NPC1 deficient microglia at P60. B. Quantitative analysis of the total volume of IBA1^+^ cells in the WMR of WT and NPC1 deficient mice. C. Quantitative analysis of the ratio between pS6R immunofluorescence intensity and the total volume of IBA1 cells in the WMR of WT and NPC1 deficient mice. Data are presented as mean ± SEM, (B) n = 4 mice/genotype, (C) n=~220 IBA1^+^cells from 4 mice. *P < 0.05, ****P < 0.0001. Scale bars: (A) 20 µm.
(TIF)

S2 Fig A. Expression of *Iba1*, *Clec7a*, and *Hif1a* mRNA by RNAScope in WT P7 and P60 WT and *Npc1*^*nmf164*^ mice. First row shows low magnified images of the dWMR at P7 and the ML at P60 from WT and *Npc1*^*nmf164*^ mice. Second and third rows shows high magnified image of insert as immunofluorescence and surfaces respectively. F. Quantitative analysis of *Iba1* mRNA total volume per cell in P7 WT, P60 WT, and P60 *Npc1*^*nmf164*^ mice. G. Quantitative analysis of *Clec7a* mRNA total volume per cell in P7 WT, P60 WT, and P60 *Npc1*^*nmf164*^ mice. H. Quantitative analysis of *Hif1a* mRNA total volume per cell in P7 WT, P60 WT, and P60 *Npc1*^*nmf164*^ mice. Data are presented as mean ± SEM, n = 3 mice/group. *P < 0.05, **P < 0.01, and ****P < 0.0001 by One-way ANOVA with post-hoc Tukey. Scale bar: (A) 30 and 5 μm.
(TIF)
