# Supplementary figures and images for: Role of mTORC1 signaling in postnatal microglia activation preceding neurodegeneration in a mouse model for Niemann-Pick disease Type C
Source: PLoS One. 2025 Sep 5;20(9):e0330437. doi: 10.1371/journal.pone.0330437 (PMC12412993; doi:10.1371/journal.pone.0330437)

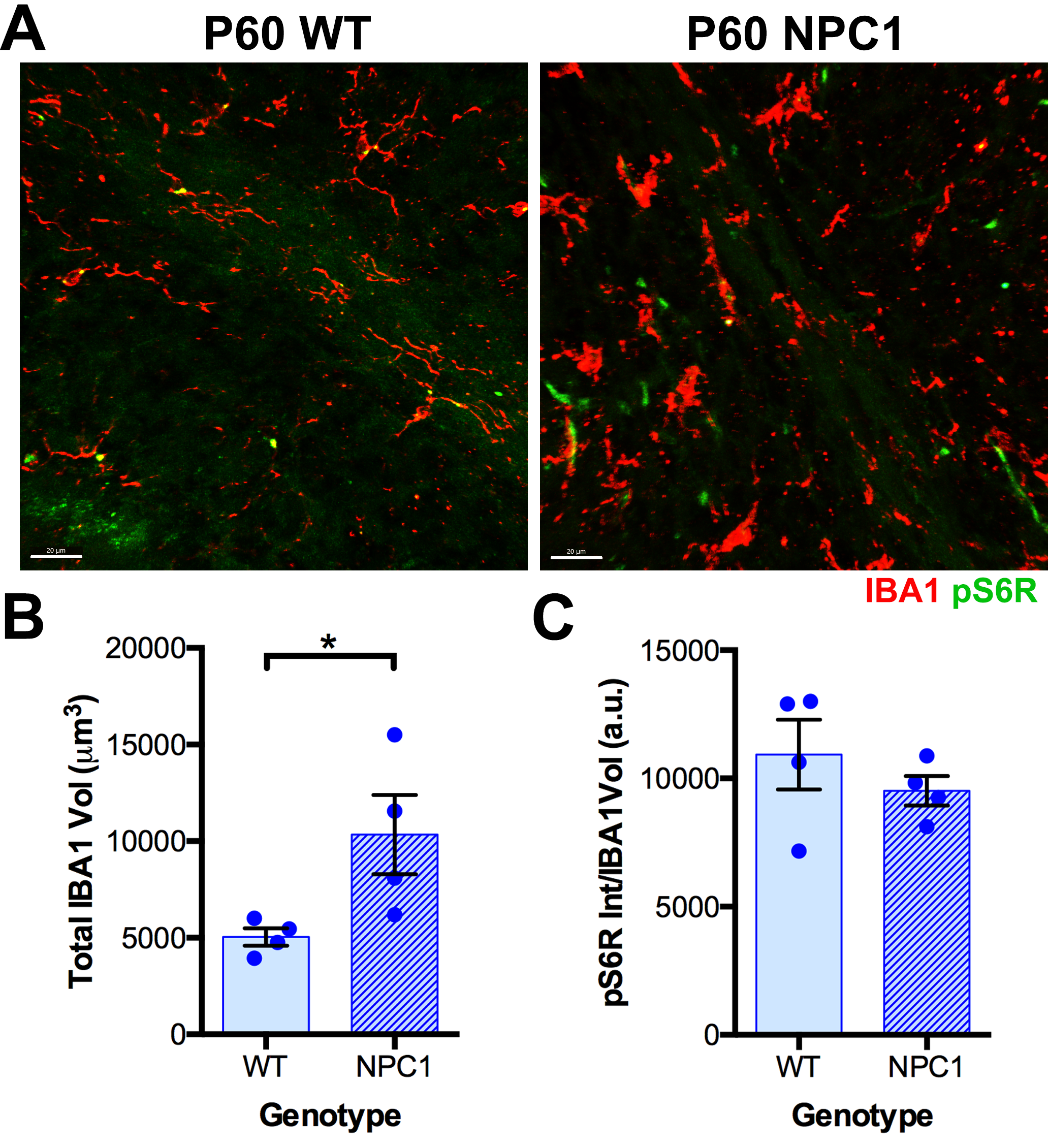

Supplement: S1 Fig — A. IBA1 and pS6R immunoreactivity in WMR microglia from WT and NPC1 deficient microglia at P60. B. Quantitative analysis of the total volume of IBA1+ cells in the WMR of WT and NPC1 deficient mice. C. Quantitative analysis of the ratio between pS6R immunofluorescence intensity and the total volume of IBA1 cells in the WMR of WT and NPC1 deficient mice. Data are presented as mean ± SEM, (B) n = 4 mice/genotype, (C) n=~220 IBA1+cells from 4 mice. *P < 0.05, ****P < 0.0001. Scale bars: (A) 20 µm. (TIF) [file pone.0330437.s001.tif]

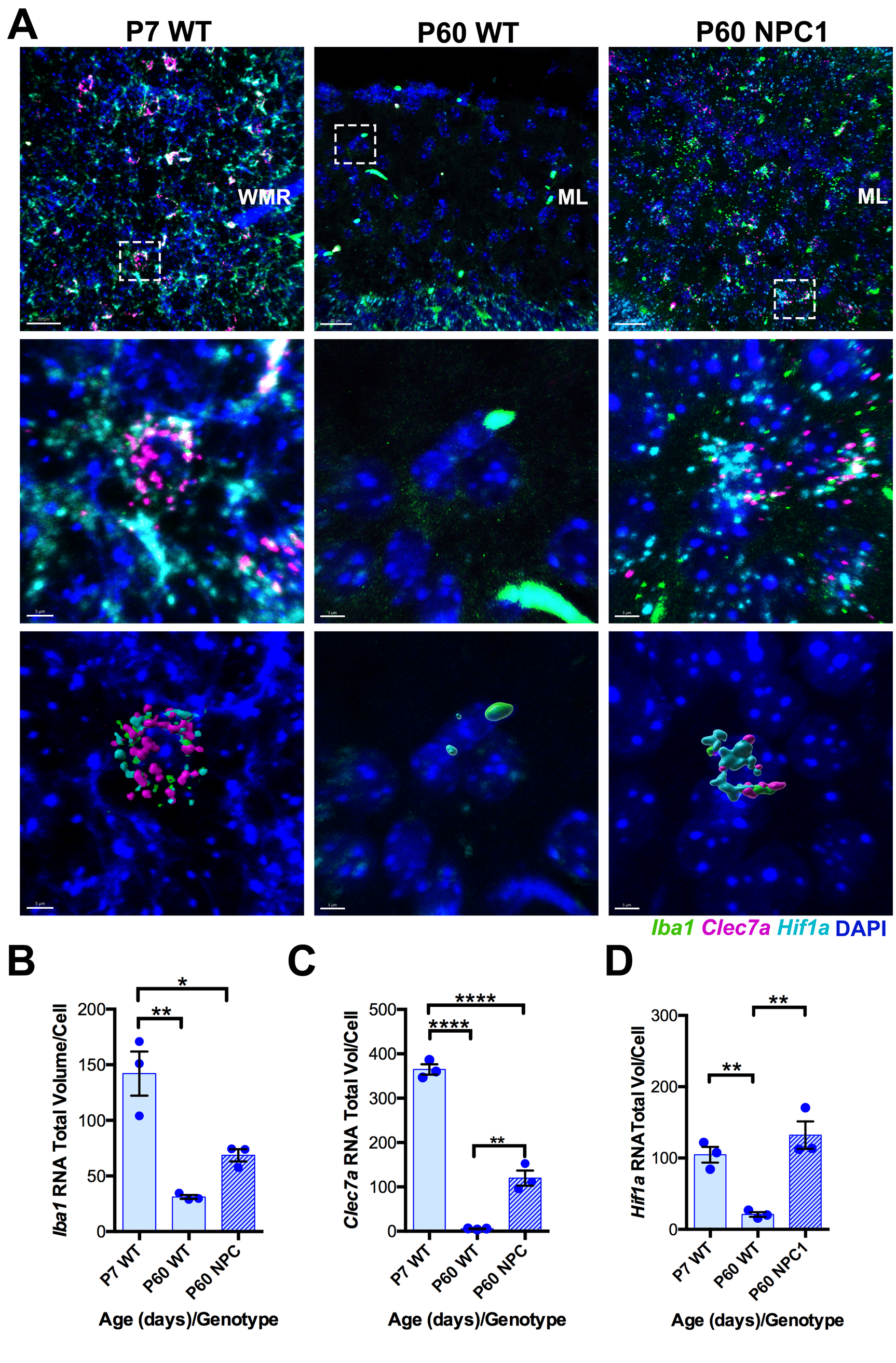

Supplement: S2 Fig — First row shows low magnified images of the dWMR at P7 and the ML at P60 from WT and Npc1nmf164 mice. Second and third rows shows high magnified image of insert as immunofluorescence and surfaces respectively. F. Quantitative analysis of Iba1 mRNA total volume per cell in P7 WT, P60 WT, and P60 Npc1nmf164 mice. G. Quantitative analysis of Clec7a mRNA total volume per cell in P7 WT, P60 WT, and P60 Npc1nmf164 mice. H. Quantitative analysis of Hif1a mRNA total volume per cell in P7 WT, P60 WT, and P60 Npc1nmf164 mice. Data are presented as mean ± SEM, n = 3 mice/group. *P < 0.05, **P < 0.01, and ****P < 0.0001 by One-way ANOVA with post-hoc Tukey. Scale bar: (A) 30 and 5 μm. (TIF) [file pone.0330437.s002.tif]
